# Two tomato GDP-D-mannose epimerase isoforms involved in ascorbate biosynthesis play specific roles in cell wall biosynthesis and development

**DOI:** 10.1093/jxb/erw260

**Published:** 2016-07-05

**Authors:** Louise Mounet-Gilbert, Marie Dumont, Carine Ferrand, Céline Bournonville, Antoine Monier, Joana Jorly, Martine Lemaire-Chamley, Kentaro Mori, Isabelle Atienza, Michel Hernould, Rebecca Stevens, Arnaud Lehner, Jean Claude Mollet, Christophe Rothan, Patrice Lerouge, Pierre Baldet

**Affiliations:** ^1^Institut National de la Recherche Agronomique (INRA), Université de Bordeaux, Unité Mixte de Recherche 1332 Biologie du Fruit et Pathologie, CS20032, F-33882 Villenave d’Ornon Cedex, France; ^2^Normandy University, Université de Rouen, Laboratoire Glycobiologie et Matrice Extracellulaire Végétale, EA4358, IRIB, VASI, 76821 Mont-Saint-Aignan, France; ^3^Institut National de la Recherche Agronomique (INRA), Unité de Recherche 1052 Génétique et Amélioration des Fruits et Légumes, Domaine Saint Maurice, 67, Allée des Chênes, CS 60094 F-84143 Montfavet Cedex, France

**Keywords:** Ascorbate, cell wall, GDP-D-mannose epimerase, growth, rhamnogalacturonan-II, *Solanum lycopersicum*, tomato fruits.

## Abstract

GDP-D-mannose epimerase (GME, EC 5.1.3.18) converts GDP-D-mannose to GDP-L-galactose, and is considered to be a central enzyme connecting the major ascorbate biosynthesis pathway to primary cell wall metabolism in higher plants. Our previous work demonstrated that GME is crucial for both ascorbate and cell wall biosynthesis in tomato. The aim of the present study was to investigate the respective role in ascorbate and cell wall biosynthesis of the two *SlGME* genes present in tomato by targeting each of them through an RNAi-silencing approach. Taken individually *SlGME1* and *SlGME2* allowed normal ascorbate accumulation in the leaf and fruits, thus suggesting the same function regarding ascorbate. However, *SlGME1* and *SlGME2* were shown to play distinct roles in cell wall biosynthesis, depending on the tissue considered. The RNAi-*SlGME1* plants harbored small and poorly seeded fruits resulting from alterations of pollen development and of pollination process. In contrast, the RNAi-*SlGME2* plants exhibited vegetative growth delay while fruits remained unaffected. Analysis of *SlGME1*- and *SlGME2*-silenced seeds and seedlings further showed that the dimerization state of pectin rhamnogalacturonan-II (RG-II) was altered only in the RNAi-*SlGME2* lines. Taken together with the preferential expression of each *SlGME* gene in different tomato tissues, these results suggest sub-functionalization of *SlGME1* and *SlGME2* and their specialization for cell wall biosynthesis in specific tomato tissues.

## Highlight

The two tomato GDP-D-mannose epimerase isoforms play specific roles in cell wall biosynthesis and plant development but participate similarly in ascorbate biosynthesis.

## Introduction

Plants exhibit variations in vitamin C or ascorbic acid (AsA) content both between plant species and in different tissues. No viable ascorbate-less mutant has ever been reported, indicating that AsA is of crucial importance in plants. AsA is a major antioxidant, which ensures the protection of plant cells against reactive oxygen species (ROS) both generated during normal physiological processes as well as by biotic and abiotic stresses. In addition, ascorbate has been implicated in the regulation of key developmental processes involving cell division and cell expansion (Smirnoff, 2000). A possible link between ascorbate and plant growth control is the interconnection between the major ascorbate biosynthetic pathway first described by Wheeler *et al.* (1998) and the cell wall biosynthesis. The formation of GDP-D-mannose is the initial step in the pathway of ascorbate biosynthesis, whilst GDP-D-mannose is also a known precursor for the synthesis of D-mannose, L-fucose and L-galactose and therefore for the pectin rhamnogalacturonan-II (RG-II) and for hemicelluloses such as (galacto)glucomannans (Reiter and Vauzin, 2001).

In addition, GDP-D-mannose epimerase (GME) produces GDP-L-galactose from GDP-D-mannose and therefore is the crossroad between L-ascorbate and cell wall polysaccharide biosynthesis. GME is the most highly conserved protein involved in ascorbate biosynthesis (Wolucka and Van Montagu, 2007) and has raised considerable interest in recent years. Change of *GME* expression may also influence ascorbate synthesis in stress conditions (Zhang *et al.*, 2011) and adjust the plant balance between ascorbate and cell wall monosaccharide biosynthesis (Wolucka and Van Montagu, 2003). Thus, it has been hypothesized that, in association with VTC2 (GDP-L-galactose phosphorylase), the enzyme catalyzing the following step of ascorbate biosynthesis, GME, constitutes a control point for the regulation of the ascorbate pathway in plants (Laing *et al.*, 2007; Wolucka and Van Montagu, 2007). Experimental evidence in support of these hypotheses are still lacking to date, possibly because knockout mutation of the two genes encoding the GDP-galactose phosphorylase VTC2 and VTC5 identified so far in Arabidopsis is lethal (Dowdle *et al.*, 2007). Two *GME* genes named *SlGME1* and *SlGME2* are present in the tomato, *Solanum lycopersicum*, genome (Zou *et al.*, 2006; Stevens *et al.*, 2007) in contrast with most plants in which *GME* exists as a single copy (Watanabe *et al.*, 2006). Analysis of tomato transcriptome data (e.g. from the tomato eFP browser, http://bar.utoronto.ca/efp_tomato/cgi-bin/efpWeb.cgi) shows that transcripts of GME1 and GME2 display specificity in their expression profiles according to the developmental stage and the organ considered (e.g. leaf, flower, fruits). Recently, Zhang *et al.* (2011) showed that the overexpression of both *SlGME1* and *SlGME2* enhanced AsA synthesis capacity and improved the tolerance of the plant to several abiotic stresses. Previously, Gilbert *et al.* (2009) demonstrated that simultaneous partial inactivation of the two *SlGME* genes in RNAi-silenced tomato lines displayed a 40–60% decrease of AsA content as well as growth defects affecting both cell division and cell expansion. Detailed analysis of the cell wall composition of these RNAi-silenced tomato lines revealed changes in the structure and the composition of hemicelluloses and pectins, as well as an alteration of the cell wall monosaccharide content, especially those directly linked to GME activity, such as D-mannose and L-galactose (Gilbert *et al.*, 2009). Subsequently it was shown that in seedlings showing a growth deficiency, the silencing of the two *Sl*GME genes resulted in a 60% decrease in terminal L-galactose in the side chain A of RG-II as well as in a lower capacity of RG-II to perform *in muro* cross-linking (Voxeur *et al.*, 2011). The restoration of both the wild-type (WT) growth phenotype as well as an efficient *in muro* boron-mediated cross-linking of RG-II by supplementation of these GME-silenced lines with boric acid and not with L-galactose or AsA strongly suggested that the growth defect in GME-deficient tomato lines was most likely related to the alteration of the D-mannose- and L-galactose-containing polysaccharides like hemicelluloses, such as mannans and the pectic RG-II, respectively, rather than to an L-AsA deficiency (Voxeur *et al.*, 2011).

Pectins are complex acidic polysaccharides of the primary cell wall containing three distinct domains: homogalacturonan, rhamnogalacturonan-I (RG-I) and RG-II (Carpita and Gibeau, 1993). RG-II is the most structurally complex pectic polysaccharide composed of a homogalacturonan backbone substituted with four structurally different oligosaccharide side chains (A–D) (O’Neill *et al.*, 2004). Despite its high complexity, RG-II represents 1–4% of the pectin-rich primary cell wall. It is evolutionary conserved as is present in the primary cell wall of all vascular plants (O’Neill *et al.*, 2004). In the primary cell wall, RG-II exists predominantly as a dimer that is cross-linked by a borate diester between two apiosyl residues of the side chain A (Kobayashi *et al.*, 1996; O’Neill *et al.*, 1996; Ishii *et al.*, 2001; Perez *et al.*, 2003). This boron-mediated cross-linking of RG-II induces the formation *in planta* of a three-dimensional pectin network that is believed to influence cell wall properties and plant growth (Funakawa and Miwa, 2015; references therein). The link between RG-II dimerization and plant development has been deduced mainly from studies of mutants affected in the RG-II biosynthesis, among them the Arabidopsis *mur1* (*murus1*) mutant deficient in the synthesis of L-fucose (O’Neill *et al.*, 2001).

In the present study, the specific silencing of each of the two GDP-D-mannose epimerases present in tomato leads to the conclusion that both *Sl*GME1 and *Sl*GME2 activities participate in the biosynthesis of AsA as they are functionally complemented in the respective RNAi-lines. Regarding the link of the GME activity with the primary cell wall metabolism, the situation is somehow different. Indeed, our data clearly showed that the two GME proteins contribute to the primary cell wall synthesis, however they strongly suggested the existence of a specialized cell wall-related activity of *Sl*GME1 and *Sl*GME2 according to the considered plant tissue. Several lines of evidence support this hypothesis and are discussed regarding the key role of GMEs in the intimate link between the metabolism of ascorbate and non-cellulosic cell wall polysaccharides.

## Materials and methods

### Plant material and culture

Cherry tomato [*Solanum lycopersicum* L. cv. West Virginia 106 (WVa106)] plants were grown in a greenhouse or *in vitro* as described (Alhagdow *et al.*, 2007). The RNAi-mediated silencing of tomato *SlGME1* (Solyc01g097340) and *SlGME2* (Solyc09g082990) genes was performed by stable transformation of tomato (Alhagdow *et al.*, 2007), using a 253bp fragment from 1238 to 1491 after the start codon (*SlGME1*) and a fragment of 285bp from 1151 to 1436 (*SlGME2*). Polyploid plants or those with multiple T-DNA insertions were excluded from further analyses. Three lines were selected as homozygous transgenic lines and kept for further studies, they correspond to L-9, L-10 and L-12 for the *P*_*35S*_ *:Slgme1* ^*RNAi*^ lines and L-7, L-10 and L-13 for the *P*_*35S*_ *:Slgme2* ^*RNAi*^ lines. Two extra *P*_*35S*_ *:Slgme1* ^*RNAi*^ lines, L-1 and L-3, were maintained as heterozygous T0 plants by cutting because they remained infertile. For *in vitro* culture, seeds of the homozygous transgenic lines and WT were treated for 10min in 3% NaOCl (v/v), washed thoroughly three times for 10min with sterile MilliQ water and sown on one-half strength Murashige and Skoog (MS) medium (Kalys-Duchefa) containing 3% (w/v) sucrose and 0.15% (w/v) Phytagel (Sigma) under a 16/8h (light/dark) photoperiod under 400 µmol m^−2^ s^−1^ light at 24 °C. For boron supplementation assays on *in vitro* seedlings, the MS medium was supplemented or not with 1.5mM boric acid. The same supplementation experiment was performed on 10-week-old plants obtained by taking cuttings from the original lines. On average six cuttings were used for each transgenic line and WT. The watering was carried out three times per week, but only once with a Liquoplant fertilizing solution (1.85g l^−1^, Plantin SARL Courthezon France) supplemented with a range of boric acid from 0.5 to 2mM and twice using tap water at pH 6.

### Flower pollination and aniline blue staining assay

The pollination was carried out by gentle vibration of the fully opened flower at anthesis, corresponding to the auto-pollination process. For the manual and the cross-pollination experiments, flower emasculation was performed on 11mm closed flowers. Then, pollen grains from flowers of the same plant or another genotype were used and thoroughly applied to the stigma by scraping the inner face of a fully opened anther cone. To ensure the cross pollination, this operation was repeated with three distinct anther cones on the same emasculated flower. The presence of pollen grains on stigma, the growth of pollen tubes in the style and surrounding the ovules inside the ovary were analyzed by aniline blue staining as previously described by Johnson-Brousseau and McCormick (2004). After 6 to 8h, the pollinated flowers were incubated in the fixing solution containing ethanol/acetic acid/water (70:5:25, v/v/v) for 2h at room temperature. The fixed flowers were sequentially rehydrated in 70%, 50% and 30% ethanol for 30min, and finally washed with distilled water three times for 5min. The rehydrated flowers were deposited on a glass slide and treated overnight in a softening solution composed of 8M NaOH, at room temperature in a wet chamber. After several washes with distilled water the pistil tissues were stained with decolorized aniline Blue solution [0.1% (w/v) aniline blue in 100mM K_3_PO_4_ buffer, pH 11] for 2h in the dark (Johnson-Brousseau and McCormick, 2004). The UV illumination was used to detect the aniline blue-stained pollen grains and tubes using an inverted Leica DMI6000B microscope equipped with a Leica DFC300FX camera.

### *In vitro* pollen germination and characterization of pollen grains

*In vitro* pollen germination was carried out according to Ye *et al.* (2009). Pollen grains were isolated from anther cones of newly opened flowers that were torn open, pinched and disseminated over a plate containing 3ml of germination medium [20mM MES pH 6, 3mM Ca(NO_3_)_2_, 1mM KCl, 0.8mM MgSO_4_, 1.6mM H_3_BO_3_, 2.5% sucrose and 24% PEG 4000]. The plate was incubated at 25 °C for 3 and 6h. Pollen grains and pollen tubes were observed using an Axioplan microscope (Zeiss). Digital images were acquired with a Moticam 2300 CCD camera (Motic Deutschland GmbH, Germany) using Motic Images Plus 2.0 software. To calculate the germination rate, a minimum of 200 pollen grains were counted in each experiment and at least three independent experiments were conducted. For defining the germination percentage, it has been considered that protrusions of the pollen tube tip from the pollen grain aperture were regarded as positive germination (Gibbon *et al.*, 1999). To estimate the pollen density presents in the stamens, one fully opened flower was vibrated three times over a microplate containing 1ml of germination medium. Pollen grains were counted using a Malassez slide and this operation was repeated with at least six flowers of each genotype.

### Histological analysis

Histological analysis was carried out according to Brukhin *et al.* (2003). Flower buds were fixed in FAA solution (4% paraformaldehyde, 50% ethanol, 5% acetic acid in 1× PBS), placed under vacuum for 10min and incubated overnight at 4 °C, dehydrated in alcohol and embedded in paraffin (Paraplast plus, Sigma). At least ten buds were sampled and 8 µm-thick sections were stained with 0.05% toluidine blue and slides were observed under a microscope (Zeiss-Axioplan).

### Ascorbate content

Mature leaves, fruits (20 DPA, red ripe) and seedlings were harvested at 12:00 am UT and thoroughly frozen in liquid nitrogen. Ascorbate content was measured according to Stevens *et al.* (2006) using 30mg FW and 100mg FW for the leaves and fruits, respectively.

### Expression analysis

RNA extraction and RT-PCR analyses using gene-specific primers (Supplementary Table S1 at *JXB* online) were performed on several tomato organs, namely leaves, fruits, seedlings and flowers for both *SlGME1* and *SlGME2* genes as previously described by Gilbert *et al.* (2009). The efficiency of the two sets of primers for GME1 and GME2 are 95% and 93.5%, respectively, making applicable the expression comparison in all the tomato organs.

### Isolation of RG-II

Dry seeds or freeze-dried seedlings were ground into liquid nitrogen and 100mg or 10mg of powder was mixed with 1ml of 70% EtOH in a 1.5ml Eppendorf, respectively. The homogenate was centrifuged at 13 000 *g* for 15min and the pellet was washed with 70% ethanol until the supernatant was clear. This step was repeated three times. The pellet was then treated overnight at room temperature and under agitation with 750 µl of a mix of methanol/chloroform (1:1). An additional washing was performed in 100% acetone for 1h. The resulting alcohol insoluble residue (AIR) was dried and weighted before being treated with 1M Na_2_CO_3_ at 4 °C for 16h and then rinsed with water to reach a neutral pH. The pellet was suspended in 500 µl ammonium acetate buffer (0.05M, pH 4.8) with 5U *endo*-polygalacturonase from *Aspergillus niger* (Megazyme, Illinois, USA) and incubated at 37 °C for 16h. The solubilized material was concentrated in a centrifugal vacuum concentrator. The RG-II was suspended in ammonium acetate buffer.

### Polyacrylamide gel electrophoresis of RG-II

Polyacrylamide gel electrophoresis was performed according to Chormova *et al.* (2014) with few changes. A 10% polyacrylamide stacking gel was added to a 26% polyacrylamide gel. 8 µl of RG-II samples (corresponding to 4–7mg and 1–1.5mg AIR for seeds and seedlings, respectively) were mixed with 2 µl sample buffer [0.63M Tris-HCl containing 0.25% (w/v) bromophenol blue and 50% (v/v) glycerol, pH 8.8]. 80 µg of RG-II extracted from lemon fruits was used as control. For monomerization, lemon RG-II was incubated for 16h at room temperature in 0.1M HCl (v/v). Before loading, the sample was neutralized with 0.1M NaOH (v/v). Electrophoresis was conducted at 220V for 130min. The gel was then fixed in ethanol/acetic acid/water (4:1:5) for 30min and washed three times in 30% ethanol for 10min and three times in water for 10min each. Silver staining was performed as described by Chormova *et al.* (2014).

## Results

### *SlGME1* and *SlGME2* display tissue- and temporal-specific expression patterns

**Fig. 1.**
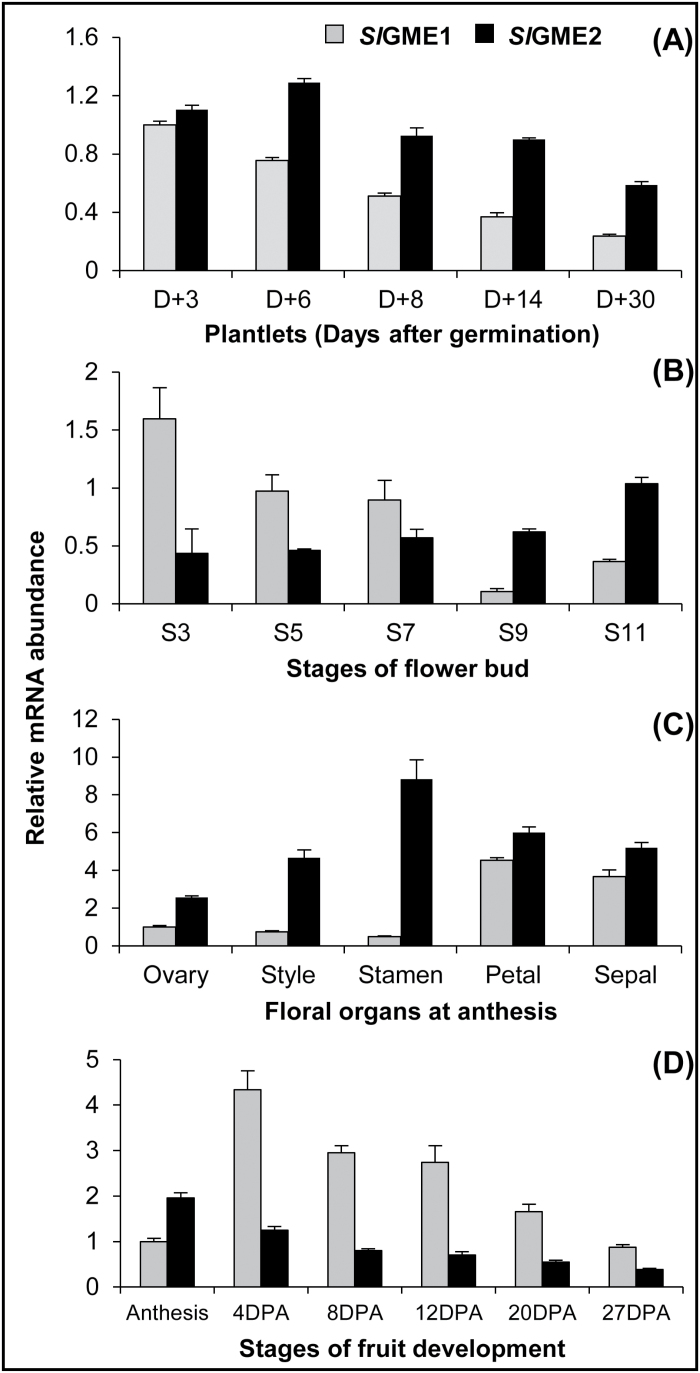
*SlGME1* and *SlGME2* expression in several organs of cherry tomato WVa106 plants. Relative *SlGME1* and *SlGME2* transcript levels in (A) plantlets during the first four weeks after germination, (B) during flower bud development from stage 3 to 11mm, (C) in several floral organs at anthesis and (D) during fruit development from fruit set (anthesis) and thereafter through the division phase at 4 and 8 DPA, and the expansion phase at 12, 20 and 27 DPA. Data are the means ±SD of three biological replicates with two technical repeats each and were obtained by quantitative RT-PCR normalized against *EIF4a*.

To extend our understanding on the respective roles of the two GME proteins in tomato plants, analysis of their expression patterns was carried out in three distinct tomato organs during their development. Firstly, during the early period of the vegetative growth after germination, *SlGME1* and *SlGME2* display distinct expression patterns, as the expression of *SlGME2* is the highest at the earlier stages (D+3, D+6) and then decreases slowly, whereas *SlGME1* expression decreases as early as D+6 and thereafter is gradually reduced up to 20% of the initial level at D+3 (Fig. 1A). In flowers the scenario is different. During the initial stages of floral bud development, *SlGME1* is 3–4-fold more expressed than *SlGME2* and then it declines progressively to reach 20% of its initial level at stage S11 just before anthesis (Fig. 1B). On the contrary, during the same developmental phase, the expression of *SlGME2* increases gradually by 2.5-fold up to stage S11. In fully opened flower at anthesis, both *SlGME1* and *SlGME2* genes display expression patterns that vary depending on the organ (Fig. 1C). Moreover, *SlGME2* expression is always higher than *SlGME1* in all the floral organs, especially in stamens, style and ovary, and at its weakest level in petals and sepals. Finally, in fruits, *SlGME1* and *SlGME2* expression is also different during the active growth phase of the tomato fruit from anthesis through the division and expansion phases (Fig. 1D). At anthesis, *SlGME2* expression is still twofold higher than *SlGME1* expression, but thereafter *SlGME2* expression declines gradually up to almost the end of the expansion phase to reach 20% of its initial expression. In contrast, after anthesis *SlGME1* expression displays a 4.4-fold increase with a peak in the middle of the division phase at 4 DPA, after which its expression decreases in parallel with *SlGME2* up to the end of the expansion phase at 27 DPA, returning to its initial value.

### SlGME1 and SlGME2 participate equally in AsA biosynthesis

**Fig. 2.**
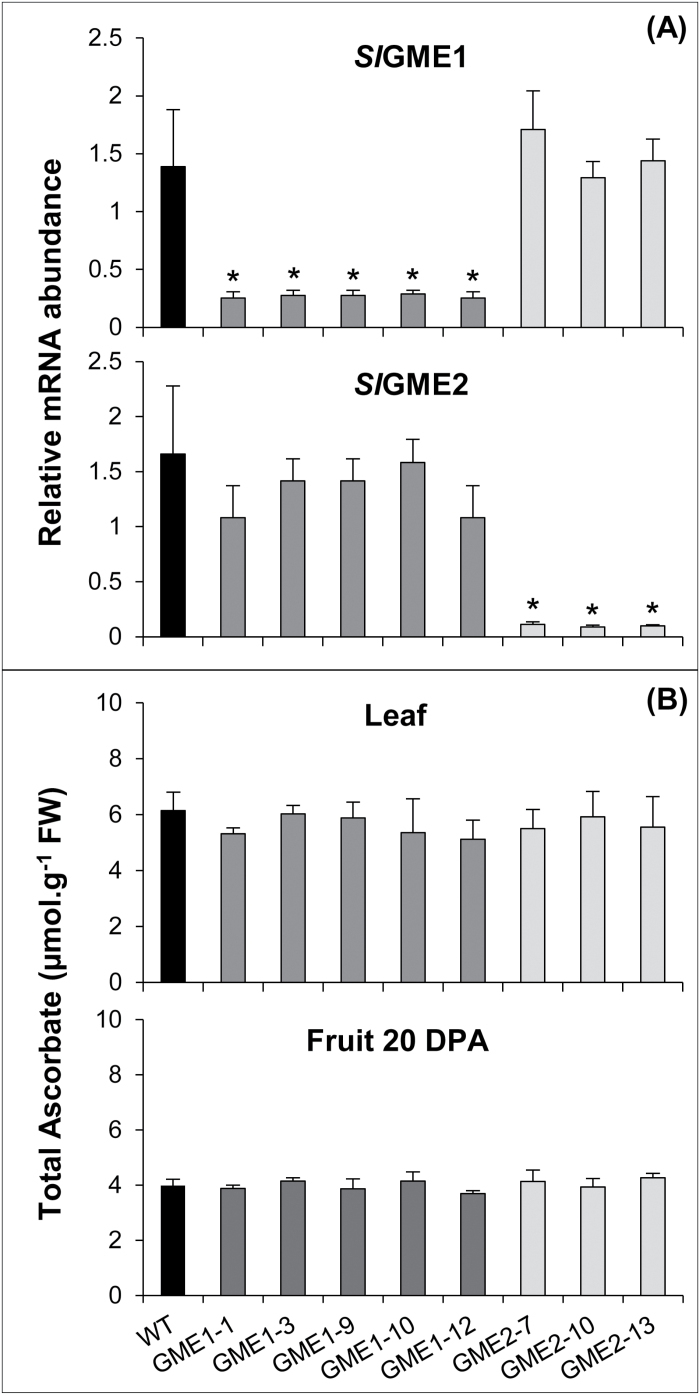
Gene expression and AsA content in *P*_*35S*_ *:Slgme1* ^*RNAi*^ and *P*_*35S*_ *:Slgme2* ^*RNAi*^ transgenic and control plants. (A) The relative abundances of *SlGME1* and *SlGME2* transcripts were determined in young leaves of *P*_*35S*_ *:Slgme1* ^*RNAi*^ (lines L-1, L-3, L-9, L-10 and L-12) and *P*_*35S*_ *:Slgme2* ^*RNAi*^ (lines L-7, L-10 and L-13) T0 plants, and control plants (WT). Data are the means ±SD of three biological replicates with two technical repeats each and were obtained by quantitative RT-PCR normalized against *EIF4a*. Asterisks indicate values that are significantly different from those of the control (*t*-test, *P*<0.05). (B) Ascorbate content in young leaves corresponding to leaflets of leaves 3, 4 and 5 below the shoot apical meristem and fruit at 20 DPA from *P*_*35S*_ *:Slgme1* ^*RNAi*^ (lines L-1, L-3, L-9, L-10 and L-12) and *P*_*35S*_ *:Slgme2* ^*RNAi*^ (lines L-7, L-10 and L-13) T0 plants, and control plants (WT). Data are the means ±SD of a total three leaves and six fruits from each transgenic line and control.

According to the distinct expression patterns of *SlGME1* and *SlGME2*, it was tempting to hypothesize that the two GME proteins play specific functions in tomato, a question all the more relevant given that tomato displays two *GME* genes (Watanabe *et al.*, 2006) whereas GME exist as a single gene in most plants studied so far. To further investigate this question, specific RNAi lines were generated for each *SlGME* gene using a specific RNA interference sequence fragment under the control of the CaMV35S promoter (named *P*_*35S*_ *:Slgme* ^*RNAi*^ transformants). From 15 independent primary *P*_*35S*_ *:Slgme1* ^*RNAi*^ and *P*_*35S*_ *:Slgme2* ^*RNAi*^ transformants (named T0) showing the presence of a single copy of the transgene, several independent lines for each gene were selected for further analyses on the T1 generation plants. However, for two lines among the selected *P*_*35S*_ *:Slgme1* ^*RNAi*^ lines – L-1 and L-3 – this was not possible as they were infertile, producing small seedless fruits. Consequently, these plants were kept and propagated as T0 plants by taking cuttings. Analysis of *SlGME* transcripts in leaves and 20 DPA fruits from the T0 *P*_*35S*_ *:Slgme* ^*RNAi*^ lines showed, as expected, that the expressions of *SlGME1* and *SlGME2* were strongly reduced in the corresponding *P*_*35S*_ *:Slgme* ^*RNAi*^ lines, leading to 20% and 5% of residual expression in *P*_*35S*_ *:Slgme1* ^*RNAi*^ and *P*_*35S*_ *:Slgme2* ^*RNAi*^ lines, respectively (Fig. 2). The silencing specifically targeted the expected *SlGME* gene since in *P*_*35S*_ *:Slgme1* ^*RNAi*^ lines the expression of *SlGME2* was not reduced and vice versa. It is noteworthy that this is also the case during flower development and to a lesser extent in developing plantlets, since no modification of the *SlGME1* expression level was observed in these *P*_*35S*_ *:Slgme2* ^*RNAi*^ lines, and vice versa (Supplementary Fig. S1). Moreover, this analysis revealed that in organs of the *P*_*35S*_ *:Slgme2* ^*RNAi*^ lines the expression of *SlGME1* was not increased, suggesting the absence of any compensatory effect for the reduction of the *SlGME2* transcript level (Fig. 2A, Supplementary Fig. S1). This observation was also true for *SlGME2* transcripts in the *P*_*35S*_ *:Slgme1* ^*RNAi*^ lines. In contrast with the previous work of Gilbert *et al.* (2009) in which the two *SlGME* genes were RNAi-silenced resulting in a 50–60% reduction of the AsA biosynthesis capacity, in the present analysis no change in AsA content was measured either in the leaf or fruits in the two types of transgenic lines (Fig. 2B) and this suggests two possibilities. First, each of the GME proteins taken alone has the capacity to maintain a WT-like AsA biosynthesis, thus corresponding to functional redundancy between GME1 and GME2. Second, together they share this capacity but even with a reduced expression of 10–20% compared to the WT, the low protein level could be sufficient to maintain the AsA biosynthesis.

### *SlGME1* silencing resulted in the reduction of fruit size

**Fig. 3.**
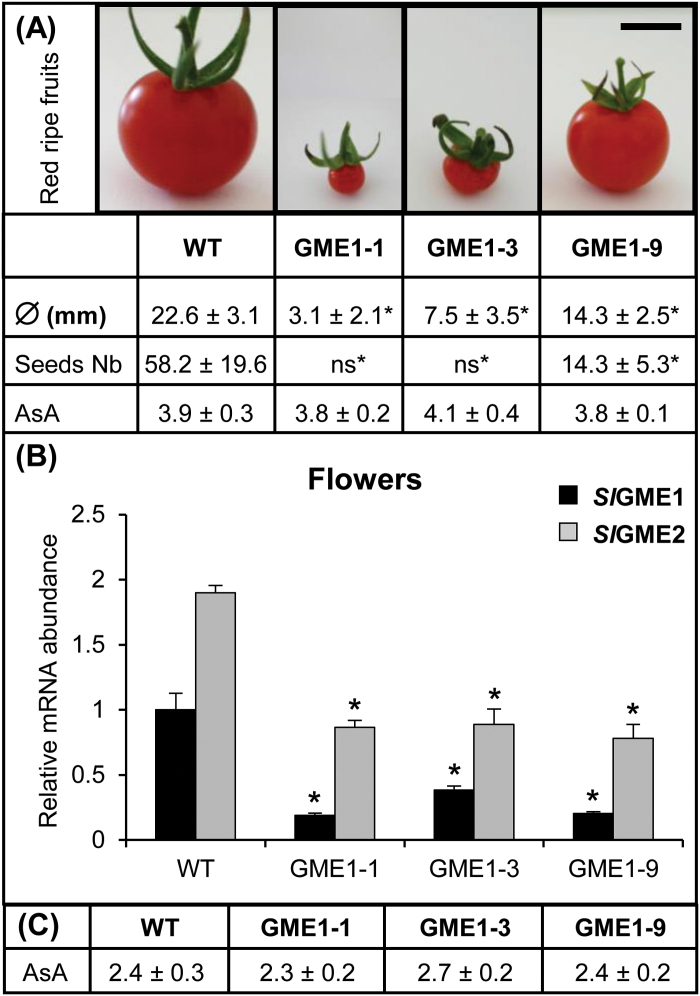
Analysis of red-ripe fruits and expression of the *SlGME* genes of flowers from *P*_*35S*_ *:Slgme1* ^*RNAi*^ lines and control. (A) Several traits of the red-ripe fruits produced by the *P*_*35S*_ *:Slgme1* ^*RNAi*^ lines L-1, L-3, L-9 and control plants were measured: fruit diameter, seed number and ascorbate concentration (expressed in µmol g^−1^ FW). Data are the means ±SD of a total of ten fruits from each transgenic line and control. Asterisks indicate values that are significantly different from those of the control plants (*t*-test, *P*<0.05); ns, infertile fruits with no seed or containing tiny seeds unable to germinate. Scale bar, 1cm. (B) The relative abundance of *SlGME1* transcripts was determined in fully developed flowers (S11) of the *P*_*35S*_ *:Slgme1* ^*RNAi*^ lines L-1, L-3, L-9 and compared to control (WT). Data are the means ±SD of three biological replicates with two technical repeats each and were obtained by quantitative RT-PCR normalized against *EIF4a*. (C) Ascorbate concentration assayed in flowers (S11). Data are the means ±SD of a total of 12 flowers from each transgenic line and control.

Among the *P*_*35S*_ *:Slgme1* ^*RNAi*^ transgenic plants, lines L-1 and L-3 exhibited severe fruit growth defects, which resulted in the development of very small and seedless fruits compared to the line L-9 and WT (Fig. 3A; Supplementary Fig. S2). In addition, AsA concentration of fully developed flowers (S11) and fruits of L-1, L-3 and L-9 remained unchanged and equal to that of the WT fruits (Figs 2, 3A–C). The more severe fruit size phenotypes observed in lines L-1 and L-3 in comparison with line L-9 were not correlated with a stronger repression of GME1 gene (Fig. 3B). In contrast, L-9, L-10 and L-12 plants were able to produce bigger fruits containing seeds but still fewer than WT fruits and their size were 30–40% smaller than fruits from *P*_*35S*_ *:Slgme2* ^*RNAi*^ lines L-7, L-12 and L-13 and the WT (Supplementary Fig. S2). The same decrease in fruit size has been described in fruits from the *P*_*35S*_ *:Slgme* ^*RNAi*^ plants in which the two *SlGME* genes were silenced (Gilbert *et al.*, 2009).

**Fig. 4.**
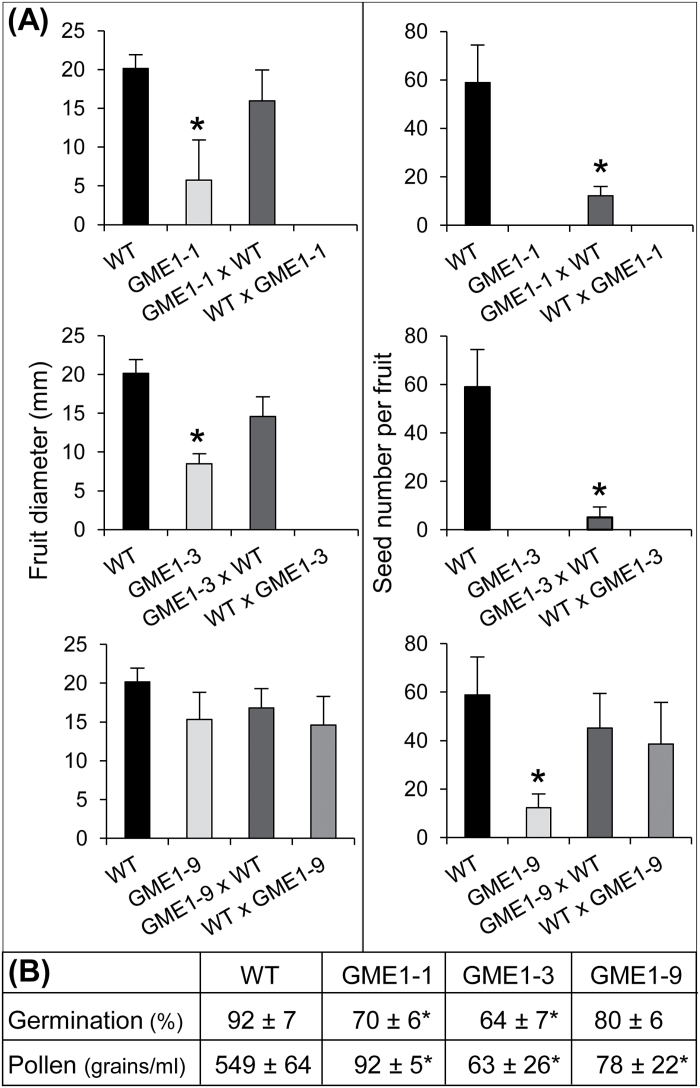
Effect of SlGME1 silencing on pollination, pollen content, pollen germination, fruit size and seed number in *P*_*35S*_ *:Slgme1* ^*RNAi*^ plants. (A) The pollination of the fully opened flower at anthesis was carried out manually as described in ‘Materials and methods’ section. Thus, three pollen cones (male part) from flowers at anthesis of the same plant or from another genotype were used on emasculated flowers (female part). In this cross-pollination method the first floral part named corresponded to the female acceptor and the second is the male donor (e.g. ‘female’ GME1-1 × ‘male’ WT). All data are the means of fruits from ten manually pollinated flowers. (B) Pollen content and germination level. Estimated pollen content per flower (*n*=10) of the *P*_*35S*_ *:Slgme1* ^*RNAi*^ line L-9, the most affected *P*_*35S*_ *:Slgme1* ^*RNAi*^ lines L-1, L-3 and the WT plants was determined as described in ‘Materials and methods’ section. The germination percentage was calculated by counting at least 200 pollen grains after 3h incubation in 3ml germination medium. Asterisks indicate values that are significantly different from those of the control (*t*-test, *P*<0.05).

Thereafter, the male or female origins of these phenotypes was investigated through cross-pollination experiments using *P*_*35S*_ *:Slgme1* ^*RNAi*^ lines L-1, L-3, L-9 and WT plants (Fig. 4A; Supplementary Table S2). It appeared clearly that the defect in fertilization originated from the male gametophyte rather than the female counterpart. Indeed, in the two extreme *P*_*35S*_ *:Slgme1* ^*RNAi*^ lines L-1 and L-3, manual pollination with their self-pollen led to the development of small fruits without seed (Fig. 4A), as observed in natural pollination conditions (Fig. 3A). When WT pollen was used to pollinate the flowers of the three *P*_*35S*_ *:Slgme1* ^*RNAi*^ lines, fruit development was restored in term of fruit size even though the seed content was still low compared to the WT (Fig. 4A). When the *P*_*35S*_ *:Slgme1* ^*RNAi*^ L-1 or L-3 line was used as pollen donor in the cross-pollination, fruit development was not restored, thus indicating a strong defect of the male reproduction organs in these *P*_*35S*_ *:Slgme1* ^*RNAi*^ lines (L-1 and L-3). In contrast, fertilization was not significantly decreased in cross-pollination experiments using L-9 moderate *P*_*35S*_ *:Slgme1* ^*RNAi*^ line as parent, except in the auto-pollination trial, which confirms also some fertilization defect in this genotype. Consistent with these results, the germination rates in comparison to the WT were significantly reduced in lines L-1 and L-3 but not in line L-9 (Fig. 4B). On the contrary, the values for pollen density differed little between these three *P*_*35S*_ *:Slgme1* ^*RNAi*^ lines, but both were very low compared to the WT (Fig. 4B). Analysis by fluorescence microscopy of the pollination process showed that the pollen tube growth was lacking in lines L-1, whereas it was normal in line L-9 (Supplementary Fig. S3), thus confirming fertilization data from Fig. 4. Finally, histological analyses of flower buds from the most affected *P*_*35S*_ *:Slgme* ^*RNAi*^ lines L-1 and L-3 clearly confirmed the very low pollen density in the pollen sacs and the high proportion of pollen grains that remained at the tetrad stage, suggesting an impaired pollen development in comparison with the WT (Supplementary Fig. S4). All these results demonstrate that silencing the *SlGME1* gene affects many aspects of the fertilization process. In both *P*_*35S*_ *:Slgme1* ^*RNAi*^ lines L-1, L-3 and L-9 this defect originates from pollen quality, quantity and its ability to germinate (Fig. 4; Supplementary Fig. S4). Additionally, the deficiency of pollen tube growth observed in the *P*_*35S*_ *:Slgme1* ^*RNAi*^ lines L-1 and L-3 may accentuate these defects and lead to the generation of very small seedless fruits (Supplementary Fig. S4). This is not the case for line L-9 where pollen tube growth is similar to the WT, and fruit growth is almost normal and like the WT (Supplementary Fig. S4). It is well known that once pollen set onto the stigma papillae, the pollen tube begins its growth through the transmitting track to reach and fecundate the ovules. This fecundation triggers an auxin burst that corresponds to fruit set and the start of seed development and fruit growth (Gillaspy *et al.*, 1993).

According to Voxeur *et al.* (2011) who demonstrated that the growth defect of the *P*_*35S*_ *:Slgme* ^*RNAi*^ plants in which the two *SlGME* genes were RNAi-silenced could be reverted by addition of boric acid at 1.5mM in the germination medium, boron supplementation trials were carried out with the most affected *P*_*35S*_ *:Slgme1* ^*RNAi*^ L-1 and L-3 lines to reverse the fruit growth phenotype. Ten-week-old plants having two to three fully developing trusses were weekly watered with the boron-supplemented nutritive solution using a range of boric acid concentrations from 0.5 to 2mM. After one month of treatment, no recovery was observed as the fruits produced never reached the size of the WT fruits whatever the boric acid concentration (data not shown). Additionally, long-term exposure to boron appeared to be toxic and resulted in plant death by defoliation, which was especially rapid at 2mM boric acid.

### *P*_*35S*_ *:Slgme2* ^*RNAi*^ lines displayed a growth defect that can be rescued by boron supplementation

**Fig. 5.**
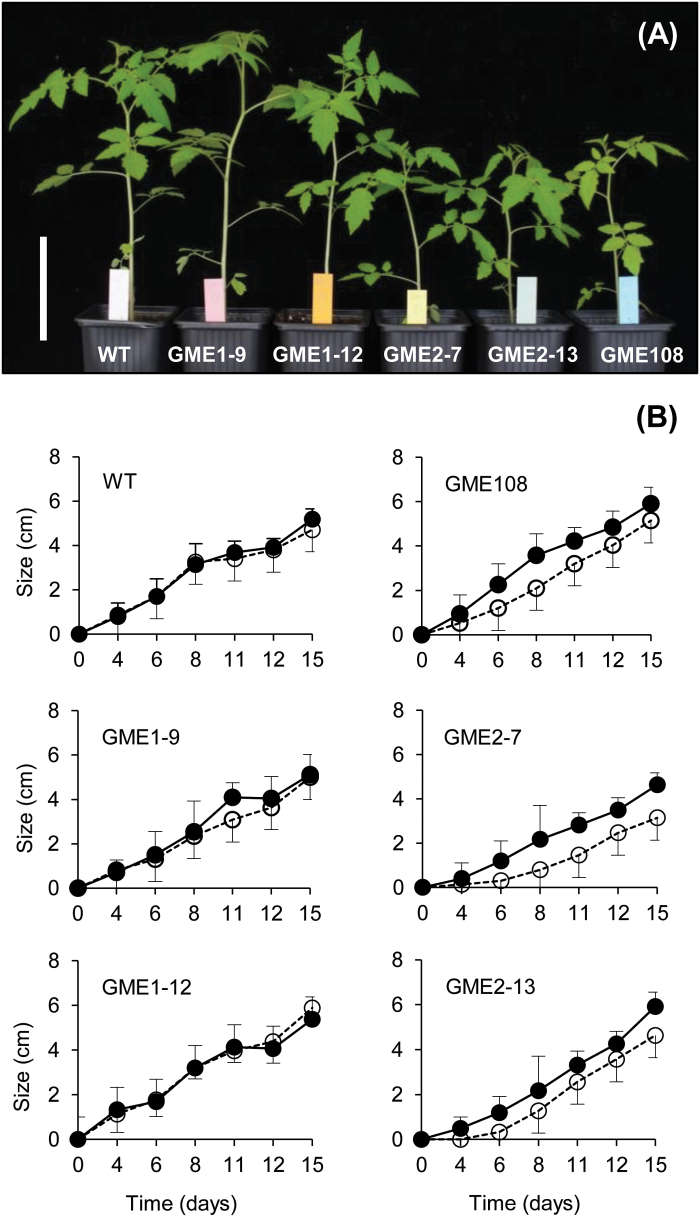
Growth of P_35S_:Slgme^RNAi^ transgenic and control plants. (A) Six-week-old WT plants, *P*_*35S*_ *:Slgme1* ^*RNAi*^ lines L-9 and L-12, *P*_*35S*_ *:Slgme2* ^*RNAi*^ lines L-7 and L-13 and the transgenic *P*_*35S*_ *:Slgme* ^*RNAi*^ line L-108 that corresponds to the RNAi-silenced line for the two *SlGME1* and *SlGME2* genes previously described by Gilbert *et al.* (2009). Scale bars, 10cm. (B) Time course of plants grown in the absence (white circle) and the presence (black circle) of 1.5mM boron from seed germination T0 up to 15 days. Data are the mean ±SD of the size of 10–12 seedlings per genotype measured in the time course.

Analysis of plant development revealed that the size of the *P*_*35S*_ *:Slgme2* ^*RNAi*^ plants after 6 weeks was reduced, whereas the *P*_*35S*_ *:Slgme1* ^*RNAi*^ plants developed similarly to the WT (Fig. 5A). The growth delay was observed very early after the germination and was maintained from then on (Fig. 5B). It is noteworthy that the growth delay of the *P*_*35S*_ *:Slgme2* ^*RNAi*^ plants was also comparable to that observed in the *P*_*35S*_ *:Slgme* ^*RNAi*^ lines (e.g. L-108) (Fig. 5; Gilbert *et al.*, 2009). As demonstrated previously, the growth defect of the *P*_*35S*_ *:Slgme* ^*RNAi*^ transformants (e.g. line L-108) originated from altered composition and structure of several cell wall polymers, including the hemicellulose mannans and the pectic RG-II (Gilbert *et al.*, 2009). Voxeur *et al.* (2011) demonstrated that boric acid supplementation during germination could revert this growth defect, notably by restoring the RG-II dimerization state. Several studies also confirmed that supplementation with boric acid may restore RG-II dimerization, cell elongation and growth (Wimmer *et al.*, 2009; Voxeur *et al.*, 2012; Smyth *et al.*, 2013). This effect of boron was also observed in the present study as 1.5mM boric acid could compensate for the growth delay of the *P*_*35S*_ *:Slgme2* ^*RNAi*^ L-7 and L-13, without affecting the growth of the *P*_*35S*_ *:Slgme1* ^*RNAi*^ and WT plantlets (Fig. 5B; Supplementary Fig. S5).

**Fig. 6.**
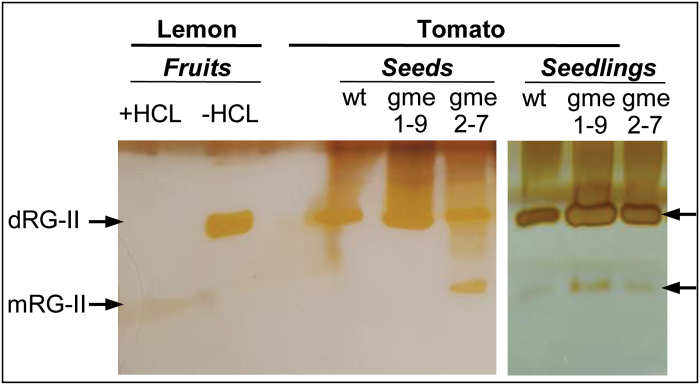
Effect of *SlGME* RNAi silencing on the RG-II dimerization detected by polyacrylamide gel electrophoresis (PAGE). Visualization of the polysaccharides was carried out by silver staining. RG-II was extracted from seeds and 4–8-day-old seedlings of WT, *P*_*35S*_ *:Slgme1* ^*RNAi*^ L-9, and *P*_*35S*_ *:Slgme2* ^*RNAi*^ lines L-7. Each lane on the gel corresponds to Alcohol Insoluble Residue (AIR) saponified and digested with the endo-polygalacturonase. RG-II dimer extracted from lemon fruits treated or not with 0.1M HCL were used as controls, mRG-II: monomeric RG-II; dRG-II: dimeric RG-II.

Consequently, the RG-II dimerization state was analyzed by PAGE in seeds and seedlings of the *P*_*35S*_ *:Slgme1* ^*RNAi*^ and *P*_*35S*_ *:Slgme2* ^*RNAi*^ lines. Alteration of the RG-II dimerization state was only visible in seeds of the *P*_*35S*_ *:Slgme2* ^*RNAi*^ line, as shown by the level of monomeric form that was significantly increased, whereas in the *P*_*35S*_ *:Slgme1* ^*RNAi*^, like in the WT, the dimeric form of RG-II remained predominant and unchanged (Fig. 6). Thereafter, in the growing seedlings, no significant RG-II dimerization change was detected in *P*_*35S*_ *:Slgme2* ^*RNAi*^ lines and PAGE patterns were similar to those of the *P*_*35S*_ *:Slgme1* ^*RNAi*^ and WT. Thus, it seems that the seed germination and growth delay observed in *P*_*35S*_ *:Slgme2* ^*RNAi*^ lines could partially originate from the alteration of the dimerization of RG-II. Finally, to investigate further to what extent *Sl*GME2 may be the major form acting during the first stages of plant growth, the analysis of the abundance of the *SlGME1* and *SlGME2* transcripts was performed in the elongation zone of the tomato hypocotyl seedlings germinating and growing under light or darkness. When seedlings were germinated in darkness the fast growth led to etiolated hypocotyls in which the expression of *SlGME1* strongly declined 4-fold, whereas that of *SlGME2* remained high and similar to that under light conditions (Supplementary Fig. S6).

## Discussion

In plants, GME is a control point between AsA biosynthesis and the non-cellulosic cell wall metabolism. This was definitively established in tomato by the previous works of Gilbert *et al.* (2009) and Voxeur *et al.* (2011). Indeed, silencing the two tomato *SlGME* genes led to a decrease in the plant capacity to synthesize AsA as well as to perform normal growth (Gilbert *et al.*, 2009). This growth phenotype resulted from defects in the structure and composition of hemicelluloses (e.g. galactoglucomannans) and more specifically in the composition and the dimerization of the pectin RG-II (Gilbert *et al.*, 2009; Voxeur *et al.*, 2011). Gene expression analysis of the two *SlGME* genes in various tomato organs (Fig. 1) further suggested their possible specialization in the tomato plants. Such specialization of *GME* could be driven by a high demand for AsA and/or GME-related cell wall precursors in a specific tissue and/or developmental stage. The present work addresses the question of the sub-functionality of the two tomato *SlGME* genes regarding these two distinct metabolisms. Towards this end, each of the two *SlGME* genes has been specifically repressed using an RNAi-silencing approach, thus allowing the study of their respective roles in AsA and cell wall biosynthesis.

### *SlGME1* and *SlGME2* share an equal prevalence in the AsA biosynthesis pathway

Repression of either *SlGME1* or *SlGME2* resulted in no change in AsA content in whole plants (Fig. 2), thus suggesting that in tomato both *Sl*GME1 and *Sl*GME2 participate in the production of AsA in all plant tissues. Recently Qin *et al.* (2015) revealed that in rice, among the three GMP genes encoding the protein that precedes GME in the Wheeler-Smirnoff pathway, only two participate in AsA biosynthesis. Moreover, each of the two GMP activities exhibits organ specificity in leaves and roots (Qin *et al.*, 2015). In the same manner, Maruta *et al.* (2008) studied the activity of phosphomannose isomerase (PMI), a step upstream to GMP in the AsA pathway. Two PMI genes exist in Arabidopsis and their expressions display differences notably according to light intensity. Only RNAi-silencing of one of the two *AtPMI* genes affected AsA content in the plants. In most tomato organs, *SlGME2* appeared as the major *SlGME* expressed gene (Fig. 1). Nevertheless, our data also showed that whatever the organ considered and the magnitude of the repression of the *SlGME1* or *SlGME2* gene expression, no compensatory change in expression of the other non-silenced *SlGME* gene could be observed. The absence of respective compensation between *SlGME1* and *SlGME2* genes strengthens the hypothesis that both *Sl*GME1 and *Sl*GME2 are involved in AsA biosynthesis and that each one is sufficient to provide enough AsA for correct functioning of all tomato organs in the conditions examined.

### SlGME1 and SlGME2 exhibit organ-specialized cell wall-related roles

Regarding the role of GME in the cell wall biogenesis, the link between AsA metabolism and growth process remains complex and its regulation is far from well established. Several studies have described a possible role of AsA during growth, including cell division and cell expansion processes (Liso *et al.*, 1988, Arrigoni *et al.*, 1989; Citterio *et al.*, 1994; Innocenti *et al.*, 1990). However, very little is known about the exact mechanisms by which AsA regulates cell growth in plants (Smirnoff, 2000; Foyer and Noctor, 2005). In 2 000, Smirnoff hypothesized a model where AsA may act as a redox buffer in the cell wall compartment in relation with apoplastic ascorbate oxidase activity and hydrogen peroxide signaling. More recently, Nunes-Nesi *et al.* (2008) proposed that the interaction between AsA and growth may involve AsA per se in combination with photosynthesis and respiration processes. Ascorbate may also participate in the crosstalk between redox and phytohormone signaling thus affecting plant growth (Considine and Foyer, 2014). However, in the present context a causal relationship between AsA and plant growth alterations must be considered with caution. Indeed, supplementation with L-galactose or L-ascorbate of tomato lines silenced for both *SlGME* genes was shown to rescue WT levels of AsA but not to restore a WT growth phenotype (Gilbert *et al.*, 2009). Moreover, Voxeur *et al.* (2011) demonstrated that supplementation of GME-silenced seedlings with boron restored WT growth, and this correlated with the recovery of the RG-II dimerization state. It is thus likely that the growth phenotypes observed in either *P*_*35S*_ *:Slgme1* ^*RNAi*^ or *P*_*35S*_ *:Slgme2* ^*RNAi*^ plants arise from a defect in cell wall biosynthesis, as previously described for SlGME-silenced plants (Gilbert *et al.*, 2009; Voxeur *et al.*, 2011).

GDP-D-mannose and GDP-L-galactose, respectively substrate and product of the GME enzyme, are not only used for L-ascorbate synthesis but also for the synthesis of non-cellulosic cell wall polysaccharides and protein glycosylation (Smirnoff, 2000). Besides, GDP-D-mannose is also a precursor of L-fucose (Reiter and Vauzin, 2001). These three nucleotide sugars are used to incorporate D-mannose, L-galactose and L-fucose into several non-cellulosic cell wall polymers (Baydoun and Fry, 1988). Several lines of evidence demonstrate that in tomato the two GME proteins participate in cell wall biogenesis. Firstly, targeted silencing of each *SlGME* gene affects plant development but not ascorbate level. In addition, growth alterations are specific to each *SlGME* gene and take place in the organ in which differential expression of the corresponding gene is maximal, i.e. *SlGME1* in developing flower and fruit and *SlGME2* in growing seedlings and etiolated hypocotyl (Figs 1, 3, 5; Supplementary Fig. S6). Secondly, no compensatory expression was observed between *SlGME1* and *SlGME2* in the respective *P*_*35S*_ *:Slgme* ^*RNAi*^ plants (Fig. 2; Supplementary. Fig. S1). Third, detailed analysis of RG-II in seeds and seedlings, in which *SlGME2* deficiency leads to noticeable growth delay, indicates that developmental alteration is linked to a defect in RG-II dimerization (Fig. 6). Altogether, these results suggest specialization of each of the two tomato *SlGME* genes in specific organs and tissues. This sub-functionalization likely occurs at the transcriptional level and takes place in tissues or conditions in which there is a high demand for cell wall precursors (Matas *et al.*, 2011; Kutschera and Niklas, 2013), e.g. pollen formation and early fruit development for *SlGME1* or hypocotyl elongation and seedling growth for *SlGME2*. The prevalence of *SlGME2* expression during dark-induced fast growth in etiolated hypocotyls strengthens this hypothesis (Supplementary Fig. S6).

### Is RG-II a key component connecting GME activity and specific organ development in tomato?

In plants, the role of the RG-II in growth as well as the role of boron in the dimerization of RG-II have been well documented (Funakawa and Miwa, 2015; references therein). Alteration of the expression of genes involved in RG-II biosynthesis was reported to impair male fertility (Delmas *et al.*, 2008; Deng *et al.*, 2010; Kobayashi *et al.*, 2011; Liu *et al.*, 2011), as well as the growth and stability of the pollen tube cell wall (Dumont *et al.*, 2014). The availability of several mutants, mainly in Arabidopsis, harboring altered cell wall composition and structure allowed the determination of the function of RG-II in plants (Funakawa and Miwa, 2015; references therein). Importantly, all the alterations of the monosaccharide composition within the side chain A that is implicated in the crosslinking of RG-II by borate have been shown to result in plant dwarfism as well as impairment of pollen development (O’Neill *et al.*, 2001, 2004; Ahn *et al.*, 2006; Delmas *et al.*, 2008, Kobayashi *et al.*, 2011; Voxeur *et al.*, 2011; Dumont *et al.*, 2014). As previously shown in Voxeur *et al.* (2011), GME deficiency leads to the decrease in terminal L-Gal content in the side chain A of RG-II as well as in a lower capacity of RG-II to perform cross-linking. Furthermore, supplementation of GME-silenced lines with boric acid was able to restore an efficient *in muro* boron-mediated cross-linking of RG-II. The characterization of several monosaccharide synthases and transferases involved in RG-II synthesis also demonstrated that boron is a key player in normal RG-II assembly through the maintenance of the RG-II structural integrity and stability (Dumont *et al.*, 2015). There is therefore an intricate link between plant growth and RG-II cross-linking, which is impaired in plants showing deficiency in L-galactose in the RG-II side chain A and/or in boron.

In the plant kingdom, the mineral boron is predominantly found in the primary cell wall associated with RG-II (Kobayashi *et al.*, 1996). During pollen development, the borate-RG-II complex is crucial for pollen tube elongation (Dumont *et al.*, 2014), and in most plant species boron is highly present in the tip of the growing pollen tube (Iwai *et al.*, 2006). Moreover, as recently suggested by Funakawa and Miwa (2015) and Dumont *et al.* (2015), boron could also act as regulator of mechanisms involved in cell growth and/or cell wall integrity-sensing. Beyond its role of physical cross-linking of the pectic network, the borate-RG-II complex might be perceived as a boron reservoir in the primary cell wall (Dumont *et al.*, 2015). Thus, any alterations in the RG-II structure and composition, especially regarding its dimerization status, may impair this sequestrated-boron reservoir and consequently could impact cell wall elongation and integrity. This could be specifically the case in rapid growth processes such as germination and hypocotyl elongation (Matas *et al.*, 2011; Kutschera and Niklas, 2013), as observed for the *P*_*35S*_ *:Slgme2* ^*RNAi*^ lines (Supplementary Figs S4–6). In *Capsicum annuum,* another species from the Solanaceae family, a study of the distribution of cell wall components using an immunological approach has revealed the preponderance of RG-II in walls of differentiating cells such as during pollen development and embryogenesis compared to proliferating cells (Bárány *et al.*, 2010). Growth rescue by boron was impossible to prove in developing fruits, but several processes including pollen development, density and germination, as well as pollen tube growth, were impaired (Fig. 4; Supplementary Fig S3, S4). The RG-II structure and dimerization within the pollen wall have not been investigated in the present study, however the results clearly demonstrate the functional role for the *Sl*GME1 protein during pollen development and fertilization, although further work is needed to define that function. Finally, it is tempting to assume that decreasing the *Sl*GME1 activity may diminish the efficiency of *in muro* boron-mediated cross-linking of RG-II during pollen development and germination, and finally fertilization.

These findings emphasize the diversity of functions of RG-II in plant development, and provide a frame to explain why tomato *SlGME* genes underwent sub-functionalization in specific tissues. Actually, no RG-II- and/or ascorbate-deficient GME mutants have been described so far in plants, presumably because strong mutations would be lethal when the plant species harbors a single *GME* gene. As shown in this study, GME plays a crucial role in cell wall and AsA metabolism in tomato plants. The availability of GME-silenced plants in which the role of GME in cell wall biosynthesis in specific plant organs and conditions can be studied independently from its role in ascorbate biosynthesis will undoubtedly prove invaluable in the future.

## Supplementary data

Supplemantary data are available at *JXB* online.

Table S1. Sets of PCR primers used to amplify specific regions of the two *SlGME* genes and the reference gene *EIF4a*.

Table S2. Flower pollination and fertilization.

Figure S1. *SlGME1* and *SlGME2* expression in plantlets and flowers.

Figure S2. Size of red-ripe fruits of *P*_*35S*_ *:Slgme1* ^*RNAi*^, *P*_*35S*_ *:Slgme2* ^*RNAi*^ lines and control plants.

Figure S3. Flower pollination, pollen tube germination and elongation.

Figure S4. Histological sections of floral buds.

Figure S5. Effect of exogenous boron supply on the growth of *P*_*35S*_ *:Slgme* ^*RNAi*^ transgenic and control plants.

Figure S6. *SlGME1* and *SlGME2* expression in hypocotyls of WT tomato seedlings.

Supplementary Data

## Abbreviations:

AsA: ascorbic acid
DPA: days post anthesis
PMI: phosphomannose isomerase
GMP: GDP-D-mannose pyrophosphorylase
GME: GDP-D-mannose-3,5-epimerase.
