# Supplementary material for: Two tomato GDP-D-mannose epimerase isoforms involved in ascorbate biosynthesis play specific roles in cell wall biosynthesis and development
Source: J Exp Bot. 2016 Jul 5;67(15):4767–77. doi: 10.1093/jxb/erw260 (PMC4973747; doi:10.1093/jxb/erw260)
Supplement: Supplementary Data [file supp_67_15_4767__index.html]

Two tomato GDP-D-mannose epimerase isoforms involve in ascorbate biosynthesis play specific roles in cell wall biosynthesis and development — Supplementary Data 

# Two tomato GDP-D-mannose epimerase isoforms involved in ascorbate biosynthesis play specific roles in cell wall biosynthesis and development

## Supplementary Data

Data files

- Supplementary\_figures\_S1\_S6\_tables\_S1\_S2.docx - Supplementary Data
