# Supplementary material for: Two tomato GDP-D-mannose epimerase isoforms involved in ascorbate biosynthesis play specific roles in cell wall biosynthesis and development
Source: J Exp Bot. 2016 Jul 5;67(15):4767–77. doi: 10.1093/jxb/erw260 (PMC4973747; doi:10.1093/jxb/erw260)

**Supplementary Data**

The following materials are available in the online version of this article.

**Table S1.** Sets of PCR primers used to amplify specific regions of the two *SlGME* genes and the reference gene *EIF4a* with the corresponding accession number and size of the amplified PCR products.

| Oligo name |  | Primer sequence (5’🡪 3’) | Accession n° | Size (bp) |
| --- | --- | --- | --- | --- |
| GME1F | Forward | ATTGGAAGCCAATCCATCTG | Solyc01g097340 | 99 |
| GME1R | Reverse | AAACCAAGAAACGCCAACAA |  |  |
| GME2F | Forward | TTGTTCGCGTGGAATGTTTA | Solyc09g082990 | 93 |
| GME2R | Reverse | GTAGGTGCAATGAGGGGATG |  |  |
| *EIF4a*F | Forward | AGTGGACGATTTGGAAGGAAG | Solyc12g095990 | 106 |
| *EIF4a*R | Reverse | GCTCCTCGATTACGACGTTG |  |  |

**Table S2. Flower pollination and fertilization.**

Pollen grains from *P_35S_:Slgme1^RNAi^* line L-1, L-3, L-9 and wild-type plants were used to cross-pollinate stigma of transgenic lines and wild-type flowers. Six hours after pollination of flowers still attached to the floral truss, flowers were harvested, fixed and stained by aniline blue. The number of flowers harbouring pollen grains on stigma papillae and the number of pollen grains on stigma of the cross-pollinated flowers for each *P_35S_:Slgme1^RNAi^* L-1, L-3, L-9 lines are represented in the table.

| **♀ Flower x ♂ Flower** | **Flowers with pollen grains on stigma** | **Pollen grains on stigma** |
| --- | --- | --- |
| GME1-1 x GME1-1 | 4/10 | 0<n<2 |
| WT x GME1-1 | 9/9 | 2<n<5 |
| GME1-1 x WT | 11/12 | >10 |
| GME1-3 x GME1-3 | 0/12 | 0 |
| WT x GME1-3 | 9/9 | 2<n<5 |
| GME1-3 x WT | 11/12 | >40 |
| GME1-9 x GME1-9 | 7/9 | >20 |
| WT x GME1-9 | 5/5 | >40 |
| GME1-9 x WT | 10/11 | >40 |

**Figure S1:** *SlGME1* and *SlGME2* expression in plantlets and flowers.


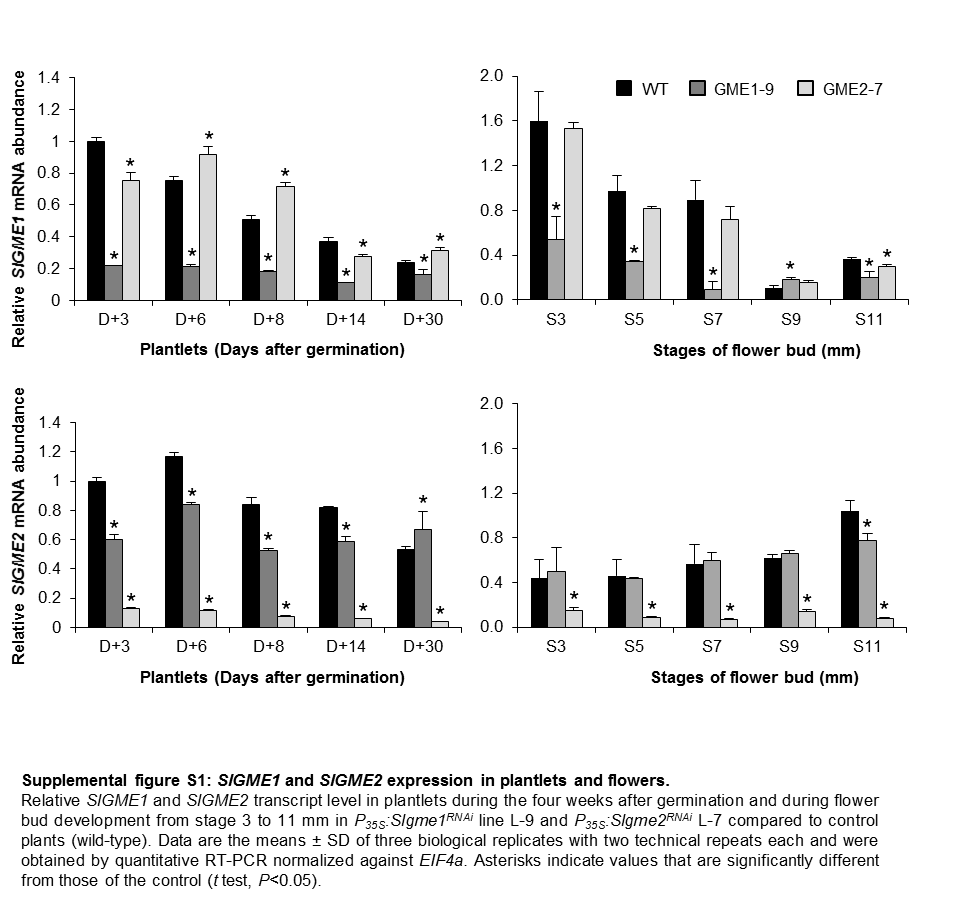


**Figure S2:** Size of red ripe fruits of *P_35S_:Slgme1^RNAi^*, *P_35S_:Slgme2^RNAi^* lines and control plants.


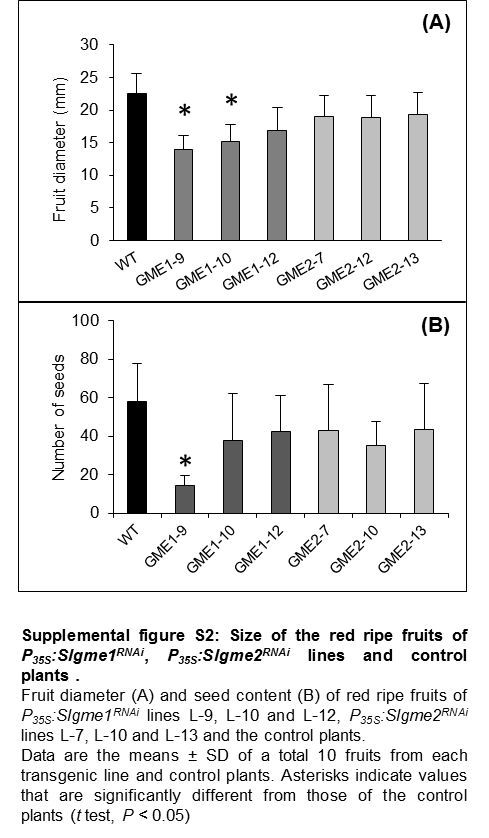


**Figure S3:** Flower pollination, pollen tube germination and elongation.


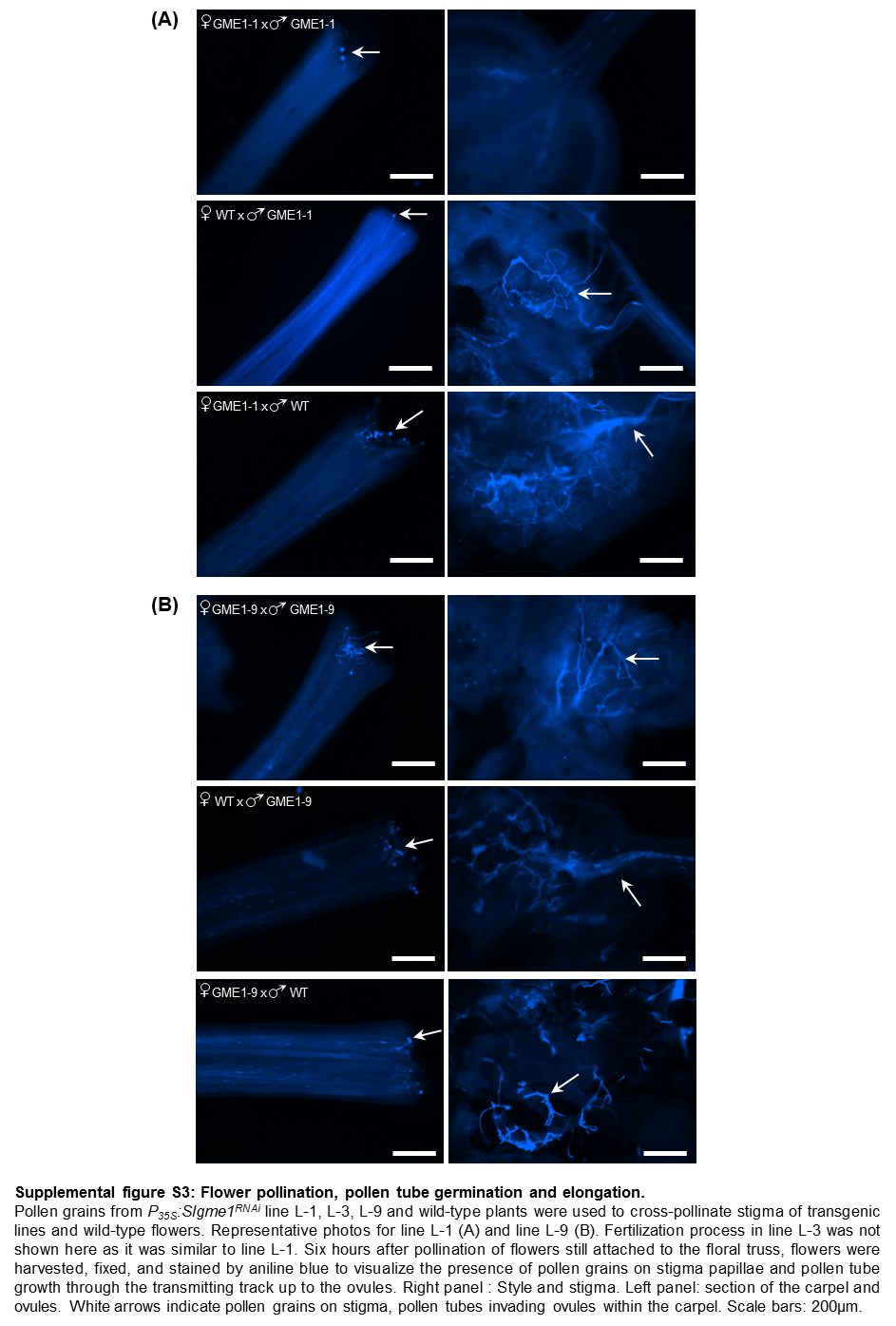
**Figure S4:**. Histological sections of floral buds.


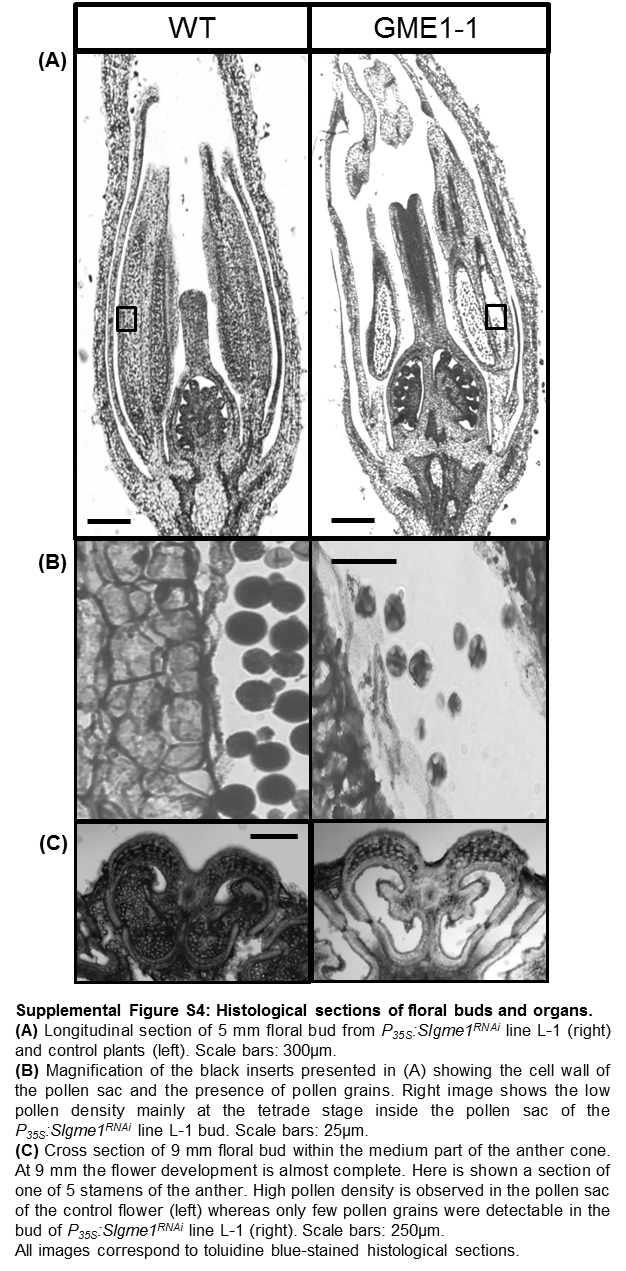


**Figure S5:** Effect of exogenous boron supply on the growth of *P_35S_:Slgme^RNAi^* transgenic and control plants


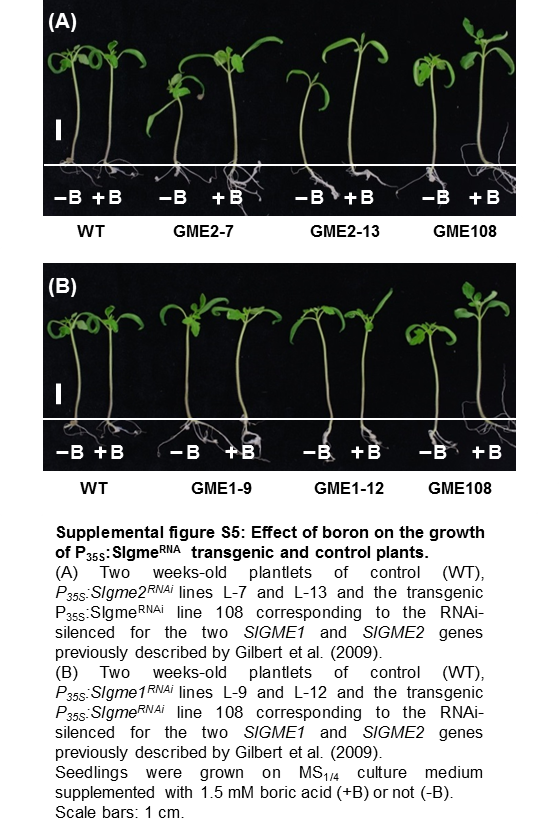


**Figure S6:** *SlGME1* and *SlGME2* expression in hypocotyls of wild-type tomato seedlings.


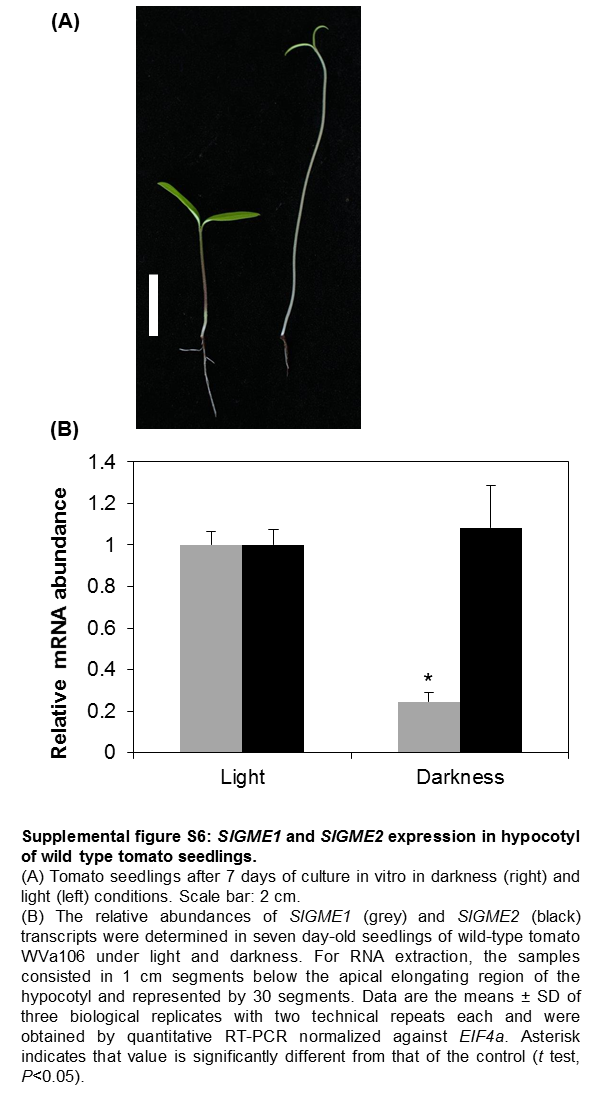

Supplement: Supplementary Data [file supp_erw260_Supplementary_figures_S1_S6_tables_S1_S2.docx]
